# Acute HSV-1 Ocular Infection Is Impaired in KLF15 Knockout Mice but Stress-Induced Reactivation from Latency Is Prolonged in Male KLF15 Knockout Mice

**DOI:** 10.3390/pathogens14080823

**Published:** 2025-08-20

**Authors:** Kelly S. Harrison, Clinton Jones

**Affiliations:** Department of Veterinary Pathobiology, College of Veterinary Medicine, Oklahoma State University, Stillwater, OK 74078, USA; hkellys@okstate.edu

**Keywords:** HSV-1, KLF15, latency, trigeminal ganglia, reactivation from latency

## Abstract

Acute human alpha-herpesvirus 1 (HSV-1) infection culminates in a latent infection of neurons in trigeminal ganglia (TG) and the central nervous system. Following infection of mucosal epithelial cells, certain neurons survive infection and life-long latency is established. Periodically, stressful stimuli trigger reactivation from latency, which result in virus shedding, transmission to other people, and, occasionally, recurrent disease. The glucocorticoid receptor (GR) and Krüppel-like factor 15 (KLF15) comprise a feed-forward transcriptional loop that cooperatively transactivate key HSV-1 promoters that drive expression of infected cell protein 0 (ICP0), ICP4, and ICP27. Silencing KLF15 significantly reduces HSV-1 replication in cultured mouse neuroblastoma cells. Consequently, we hypothesized that KLF15 mediates certain aspects of reactivation from latency. To test this hypothesis, we compared HSV-1 replication in KLF15^−/−^ mice versus wild-type (wt) parental C57BL/6 mice. Virus shedding during acute infection was reduced in KLF15^−/−^ mice. Male KLF15^−/−^ mice shed higher titers of virus during late stages of reactivation from latency compared to KLF15^−/−^ females and wt mice regardless of sex. At 15 d after explant-induced reactivation, virus shedding was higher in male KLF15^−/−^ mice relative to wt mice and female KLF15^−/−^ mice. These studies confirm KLF15 expression enhances viral replication during acute infection and reactivation from latency.

## 1. Introduction

Human alpha-herpesvirus 1 (HSV-1) infection of facial mucous membranes culminate in lifelong latency in sensory neurons in trigeminal ganglia (TG), brainstem neurons, and other neurons in the central nervous system [1,2,3]. These infections can lead to recurrent disease, including gingivostomatitis [4,5], herpes labialis [6], and stromal keratitis, which is the leading cause of infectious blindness in the world [7,8,9,10]. Furthermore, HSV-1 is the most common cause of sporadic, fatal encephalitis, in which virus reactivation from latency accounts for over two-thirds of all cases [11,12,13,14]. Commercially available HSV-1 vaccines are not available, and current antiviral drugs (acyclovir and related compounds) do not significantly reduce reactivation from latency [15,16,17].

During productive HSV-1 infection, robust viral DNA replication and protein expression occurs, which culminates in high levels of infectious progeny for virus transmission and spread of disease [18,19,20]. In latently infected neurons, the locus that encodes the latency-associated transcript (LAT), micro-RNAs, and other non-coding RNAs are the only abundantly expressed viral products during latency [21,22,23,24]. LAT gene products inhibit apoptosis and viral gene expression, thereby establishing and maintaining a reservoir of latently infected TG neurons, as reviewed in [23,24]. During latency, infectious virus is not readily detected.

Periodically, various stimuli [19,25], including physical and psychological stress, trigger reactivation from latency, as reviewed in [25,26]. Stress leads to increased corticosteroid levels that bind and activate the glucocorticoid receptor (GR), which subsequently enters the nucleus, binds specific GR response elements in DNA, and induces gene expression [27]. The synthetic corticosteroid dexamethasone (DEX) consistently induces HSV-1 virus shedding during explant-induced reactivation from latency [28,29,30]. Stress also induces expression of certain cellular transcription factors, including several members of the Krüppel-like factor (KLF) family of transcription factors, including KLF15 [31,32,33]. Notably, KLF15 plays a key role in amino acid, lipid, and glucose metabolism [34], catabolic metabolism of cardiomyocytes [35], adipogenesis [36], and maintenance of smooth muscle through interactions with GR [37,38]. Studies from our lab demonstrated GR and KLF15 cooperatively transactivate key HSV-1 immediate early promoters that drive expression of transcriptional viral regulatory proteins, including infected cell protein 0 (ICP0) [39,40], ICP4 cis-regulatory module [41], and an ICP27 cis-regulatory module [42] using transient transfection studies. Finally, silencing KLF15 impaired HSV-1 and bovine alpha-herpes virus 1 replication in permissive cultured cells [43].

Based on these observations, we hypothesized that KLF15 plays a role during the HSV-1 latency and reactivation cycle. KLF15-knockout (KLF15^−/−^) mice were infected with HSV-1 to test this hypothesis. Notably, virus shedding during acute infection was reduced in male and female KLF15^−/−^ mice relative to wild-type (wt) age-matched littermates. In contrast, when latently infected TG are explanted in medium that contains DEX to stimulate reactivation, male KLF15^−/−^ mice shed higher titers of virus at late stages of reactivation when compared to wt male and female littermates, and female KLF15^−/−^ mice. Collectively, these results revealed that KLF15 is important but not required for HSV-1 acute infection following ocular infection of mice. Furthermore, sex-specific factors regulate virus production in KLF15^−/−^ mice during explant-induced reactivation from latency.

## 2. Materials and Methods

### 2.1. Viruses and Cell Lines

Monkey kidney cells (Vero ATCC CCL-81) were grown in minimal essential medium (MEM; Corning; Life Sciences, Union City, CA, USA) supplemented with 10% FBS (Atlas Biologicals; Fort Collins, CO, USA), 2 mM L-glutamine (Corning: Life Sciences, Union City, CA, USA), and antibiotics (100 IU/mL penicillin, 100 µg/mL streptomycin, Cytiva Hyclone; Wilmington, DE, USA) at 37 °C and 5% CO_2_. HSV-1 strain McKrae was obtained from the late Dr. Steven Wechsler (University of California, Irvine Medical School) and grown in Vero cells until an >80% cytopathic effect was observed. Cells and supernatant were collected and serial freeze–thawed at 37 °C/−80 °C followed by centrifugation to remove cellular debris and then titered on Vero monolayers to determine plaque-forming unit/mL (PFU/mL). Stocks were aliquoted and stored at −80 °C prior to mouse infections.

### 2.2. Mouse Breeding and Infection Studies

All animal experiments were approved and performed in accordance with the Oklahoma State University Institutional Animal Care and Use Committee (protocol VM-24-58) and maintained in Techniplast Blue Line cages. Individually ventilated cages received 10–15 air changes/h within a 12 h light/dark cycle and an air-conditioned, temperature-, and humidity-controlled environment. Male and female mice heterozygous for KLF15/LacZ expression were kindly gifted by Dr. Anthony N. Gerber (National Jewish Health, Denver, CO, USA) [44] and fed a high-fat diet (11% LabDiet, St. Louis, MO, USA) for enhanced fertility and reproduction [45]. Upon arrival, mice were paired for breeding while under quarantine for health testing and retained until either 5 months of age or 5 successful litters (gestational period ~21 days). Ten days after birth, pups were sexed, ear-tagged, and had <2 mm of tail snipped, incubated with alkaline lysis buffer (25 mM NaOH/0.2 mM EDTA) at 98 °C for 1 h, and then neutralized with 40 mM Tris HCl (pH 5.5) [46]. Samples were centrifuged at 2000 g for 1 min to pellet debris and supernatant isolated for PCR-based genotyping. Standard endpoint PCR was used with primers designed to amplify the mouse KLF15 and LacZ insert. The KLF15 forward was 5′-GCATCTGCTGTCCACCTATT-3′; The KLF15 reverse was 5′-GTTCGTCTATGCCTCACCTATTC-3′; the LacZ forward was 5′-ATCCTCTGCATGGTCAGGTC-3′; and the LacZ reverse was 5′-CCGTGGCCTGATTCATTCC-3′. Homozygous KLF15^−/−^ male and female pups were weaned from parental breeding pairs 21 days after birth and used for subsequent breeding schemes. Between 6- and 8-weeks of age, KLF15^−/−^ littermates were divided into disposable Techniplast cages (5 mice/cage) within a BSL-2 approved facility, and C57BL/6 age-matched control animals were purchased from Jackson Labs (strain 000664; Bar Harbor, ME, USA). All mice were acclimated for a minimum of 7 days prior to infection. Mice were ocularly infected in both eyes without scarification using 10^5^ PFU of HSV-1 strain McKrae, as previously described [28,47,48]. All infection studies consisted of a minimum of two independent experiments with 4–5 mice/group unless otherwise indicated.

### 2.3. Infection of Primary Mouse Kidney Cells

Kidneys were aseptically dissected from 6–8-week-old uninfected KLF15^−/−^ and wt C57BL/6 male and female mice, and single cells were prepared as previously described [48]. Briefly, kidneys were dipped in 100% ethanol, minced into <3 mm pieces, and incubated with 0.25% trypsin for 4–18 h with rocking at 4 °C. At 4 h intervals, supernatant was removed, and single cells were pelleted via centrifugation. Remaining tissues were incubated with residual trypsin and incubated at 37 °C for 30 min. Cells were pelleted via centrifugation at 200 g for 10 min then gently dispersed by pipetting. All cells were filtered through a 100 µm cell strainer, and viability was determined via trypan blue assay. Approximately 10^5^ cells were plated per well into 24-well plates and infected with HSV-1 at a multiplicity of infection of 1 at 37 °C and 5% CO_2_ with rocking. The virus was removed and cells were incubated for 24 h prior to plaquing for virus titers on Vero cells, as described above.

### 2.4. Ocular Swabs, TG Plaque Assays, and Explant-Induced Reactivation

Swabs from cornea and conjunctiva were collected from individual animals into 1 mL MEM with L-glutamine, antibiotics, and 10% FBS every other day for the first 10 days post-infection, and every 5 days during the remainder of the experiment. Samples were frozen at −80 °C until they were ready for use. Serial dilutions of ocular swabs were used to infect Vero monolayers for 1 h at 37 °C and 5% CO_2_ with rocking. Monolayers were overlayed with 1% methylcellulose in MEM with L-glutamine, 10% FBS, and antibiotics, and monitored for visible plaques. If plaques were not observed after 72 h, samples were considered negative for the virus.

At 4- and 8-days post-infection, TG were dissected, minced into three to four pieces, and placed in 60 mm dishes with confluent Vero monolayers. If a cytopathic effect was not observed, tissue and supernatant were removed and 1% methylcellulose in MEM with L-glutamine, 10% FBS, and antibiotics was overlayed onto the monolayer and allowed to incubate for 3 days prior to crystal violet staining for plaque visualization. If a cytopathic effect was detected within 24 h, the cells and supernatant were collected, serially frozen/thawed (−80 to 37 °C), and titered on Vero monolayers.

For explant-induced reactivation, TG were collected in MEM with L-glutamine, antibiotics, 2% charcoal-stripped FBS, and 10 µM water-soluble DEX (Sigma; catalog no. D2915; St Louis, MO, USA) to induce virus reactivation from latency at 30 dpi, before being incubated in a CO_2_ incubator set at 37 °C and 5% CO_2_. Aliquots of supernatant were removed daily and used to plaque for the infectious virus on Vero monolayers, as previously described [28,47,48].

### 2.5. Reverse Transcription and Quantitative PCR (qPCR)

At the designated times, TG and kidneys were prepared. Kidneys do not contain any HSV-1 DNA following ocular infection and serve as an internal reference tissue. The use of an internal reference tissue within the same animal as experimental samples provides a twofold reduction in technical variation and PCR efficiency [48,49,50]. Tissues were minced and incubated in alkaline lysis buffer, as described for tail snips above. Lysis buffer was neutralized with 40 mM Tris-HCl pH 5 and briefly centrifuged to remove cell debris. Following cell lysis, DNA purification was performed as described [47,48,49,50]; briefly, two rounds of phenol–chloroform–isoamyl alcohol (25:24:1) extraction was performed followed by one round of chloroform–isoamyl alcohol (24:1 *v*/*v*) and ethanol precipitation. Two volumes of ice-cold 100% ethanol were added and incubated at −20 °C overnight. DNA was pelleted via centrifugation (18,000× *g* for 30 min), washed twice with 100% ethanol twice and air-dried prior to dissolving in DEPC-water. A total of 10 ng of genomic DNA was used as a template for Sybr Green qPCR (Applied Biosciences; St Louis, MO, USA) using primers for HSV-1 gB and mouse GAPDH. The gB forward primer was 5′-AACGCGACGCACATCAAG; the gB reverse primer was 5′-CTGGTACGCGATCAGAAAGC; the GAPDH forward primer was 5′-CATCACTGCCACCCAGAAGACTG; the GAPDH reverse primer was 5′-ATGCCAGTGAGCTTCCCGTTCAG.

For RT-qPCR, TG and kidney were dissected at 30 dpi and homogenized in Trizol using GentleMACS M-tubes (Auburn, CA, USA) and program RNA_01. M Tubes were centrifuged at 2000 g for 5 min prior to application with the Qiagen RNeasy Kit according to the manufacturer’s instructions. Samples were eluted with DEPC-water and analyzed for yield and quality. Here, 100 ng of RNA was used with the SuperScript III Platinum One-Step qRT-PCR Kit (Invitrogen; Carlsbad, CA, USA) according to manufacturer’s instructions using primers for HSV-1 LAT and mouse GAPDH. The LAT forward primer was 5′-CCTTATCTAAGGGCCGGCTG-3′; the LAT rev primer was 5′-GGGACACATGCCTTCTTGGA-3′.

All primers were designed, synthesized, and purchased from IDT. A BioRad CFX Opus 96 PCR system was used along with CFX Maestro Analysis Software vs 2.3; Cq values between 20 and 35 were considered positive. Ratios of gB or LAT to GAPDH were calculated using the delta–delta CT method.

### 2.6. Statistical Analysis

All graphs and comparisons were performed using GraphPad Prism vs 10.4.1 software (v10.5.p). *p* values less than 0.05 were considered significant for all calculations, with specific tests and post hoc analysis indicated in the respective figure legends.

## 3. Results

### 3.1. Generation and Validation of KLF15 Knockout Mice

**Figure 1:**
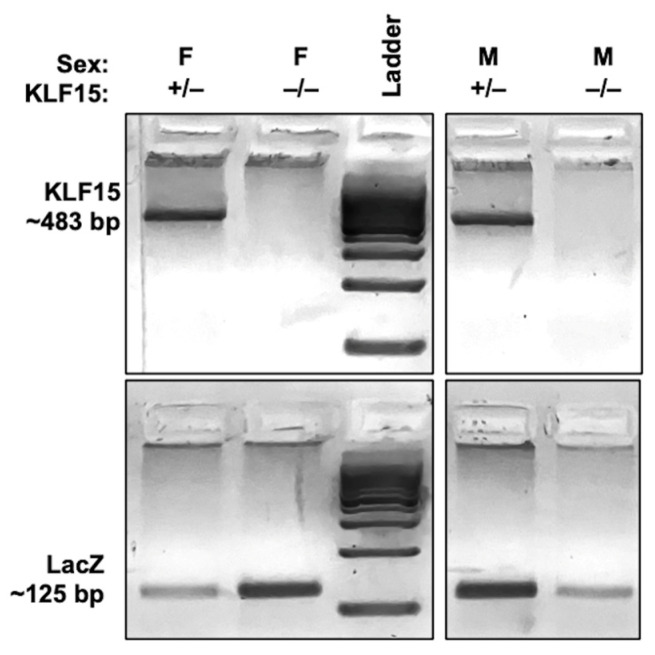
Identification of homozygous KLF15 knockout mice. Male and female mice heterozygous for KLF15/LacZ expression (KLF15^+/−^ lanes 1 and 4) were paired for breeding during and after they were quarantined for health testing. Pups were ear-tagged at postnatal day 10 and tail snips were obtained for genotyping, as discussed in the Materials and Methods section. Male and female KLF15^−/−^ pups positive only for LacZ (lanes 2 and 5) were used for breeding and infection studies.

To investigate the role KLF15 plays during HSV-1 infection, a mutant mouse model with a targeted deletion of the murine KLF15 gene was used. Heterozygous KLF15 knockout mice (KLF15^+/−^) contain a lacZ gene at the KLF15 ATG [34] and were bred to generate homozygous knockout (KLF15^−/−^) offspring. Endpoint PCR-based genotyping was performed on 10-day old pups to distinguish heterozygous from homozygous littermates using primers specific to the murine KLF15 and LacZ, as described in the materials and methods. As shown in Figure 1, KLF15 alleles were detected as ~483 bp bands (Figure 1: lanes 1 and 4), while the LacZ insertion yielded a 125 bp product (Figure 1: lanes 2 and 5). Pups expressing both genes are consistent with heterozygosity and were humanely euthanized; pups expressing only the 483 bp band (KLF15^+/+^) are referred to as wt. KLF15^−/−^ mice lack the KLF15 PCR product but contain the 125 bp LacZ band, confirming successful deletion of KLF15. These mice were used for subsequent breeding and infection studies. Notably, breeding KLF15^−/−^ males to KLF15^−/−^ females resulted in runted pups relative to wt age-matched and heterozygous KLF15^+/−^ mice. KLF15^−/−^ mouse litters weighted ~8 g by 21 days of age: consequently, weaning was delayed until 28 days after birth. WT age-matched mice weighed between 10 and 12 g and were weaned at the standard 21 days after birth. Each KLF15^−/−^ litter contained approximately four males for every female, and litters tended to be smaller.

### 3.2. Examination of HSV-1 Replication in Primary Kidney Cells from KLF15^−/−^ Mice

**Figure 2:**
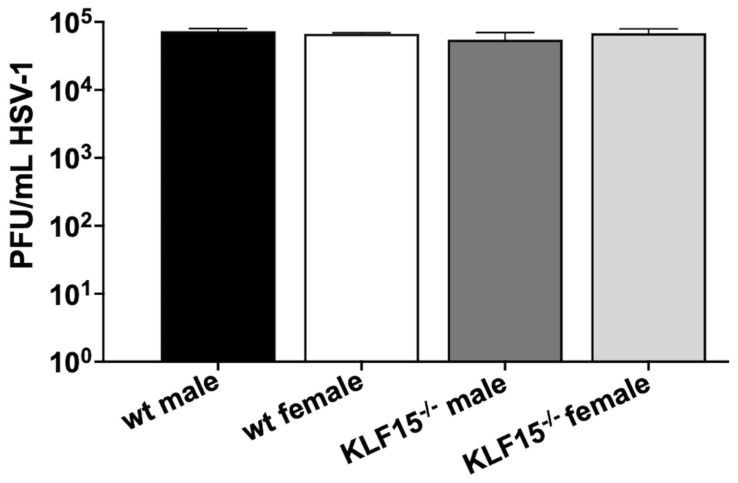
HSV-1 infection of KLF15^−/−^ primary kidney cells. Primary kidney cells were prepared from 8-week-old male and female wt and KLF15^−/−^ mice (n = 4–6 from 2 separate experiments). HSV-1 was used to infect these cells at a MOI of 1. Twenty-four hours post-infection, cells and supernatant were collected, freeze–thawed three times at 37 °C/−80 °C and subsequently used for plaque assays on Vero cell monolayers. Data shown are the mean ± SD for triplicate wells of duplicate experiments in plaque-forming units (PFU)/mL.

Primary cells were prepared from kidneys dissected from uninfected KLF15^−/−^ mice or age-matched wt controls. The neurovirulent HSV-1 strain McKrae was used to infect these cells at a multiplicity of infection (MOI) of 1. Twenty-four hours post-infection (hpi), virus replication was measured using plaque assays (Figure 2). For all groups, infection of primary kidney cells resulted in ~10^5^ plaque-forming units (PFU)/mL. There were no differences detected between sexes (male versus female) or kidney cells from wt vs. KLF15^−/−^ mice.

These data indicated that KLF15 is not required for HSV-1 replication in kidney cells. These results were different compared to previously published studies because silencing KLF15 in a mouse neuroblastoma cell line (Neuro-2a) resulted in an approximately 2-log reduction in HSV-1 virus production [43]. We suggest that KLF15 is not important in certain cell-types because other transcription factors compensate for the deletion of KLF15. For example, the ICP4 CRM was efficiently transactivated by GR and KLF15 in Vero cells but not in Neuro-2A cells [41].

### 3.3. HSV-1 Replication Is Impaired During Acute Infection of KLF15^−/−^ Mutant Mice When Compared to wt Mice

**Figure 3:**
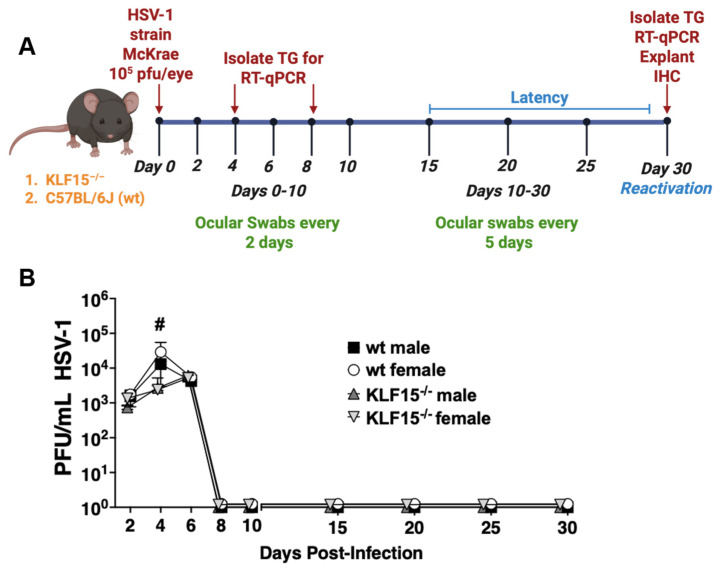
Quantification of HSV-1 virus shedding in ocular surfaces of KLF15^−/−^ and wt mice. (**A**): Schematic of animal infection studies. KLF15^−/−^ or wt mice were ocularly infected with HSV-1 on day 0. Ocular swabs were collected every other day until day 10, after which swabs were collected every 5 days. (**B**): Following ocular swabs of each group, infectious virus was measured at the denoted days after infection. Data are shown as the time-plot mean ± SEM of individual animals (n = 5 mice/group, 2 separate experiments); ^#^ *p* < 0.05 for wt mice compared to KLF15^−/−^ mice using an unpaired *t*-test.

Eight week-old KLF15^−/−^ or wt mice (males and females) were ocularly infected with HSV-1 strain McKrae, as described in the Materials and Methods section. Swabs from the orbital cavity were collected every other day for the first 10 days to measure acute virus shedding and every 5 days from days 10 through 30 to test whether there were differences in the length of acute infection (Figure 3A). Viral titers from the cornea and conjunctival surfaces were quantified using plaque assays. All groups of mice were shedding ~10^3^ PFU/mL by 2 days post-infection (dpi, Figure 3B). Virus shedding from male and female wt mice peaked at 4 dpi with ~5 × 10^4^ PFU/mL present in swabs. In contrast, virus shedding from ocular surfaces of KLF15^−/−^ mice was significantly lower at 4 dpi compared to wt mice. For example, 2500 PFU/mL were present in swabs from male and female KLF15^−/−^ mice at 4 dpi. At 6 dpi, virus shedding in ocular surfaces of wt male and female mice was reduced and was not significantly different than KLF15^−/−^ mice regardless of sex. Interestingly, HSV-1 virus shedding (~5000 PFU/mL) peaked at 6 dpi in KLF15^−/−^ male and female mice. In contrast, the peak of HSV-1 shedding in ocular swab occurred at 4 dpi. As expected, the infectious virus was not detected in ocular swabs of wt or KLF15^−/−^ mice from 8 through 30 dpi, indicating that latency was established and maintained. Mortality did not occur following ocular infection with the MOI used for these studies in wt or KLF15^−/−^ male and female mice.

### 3.4. Comparison of Infectious Virus and Total Viral DNA in TG of wt Versus KLF15^−/−^ Mice After Infection

**Figure 4:**
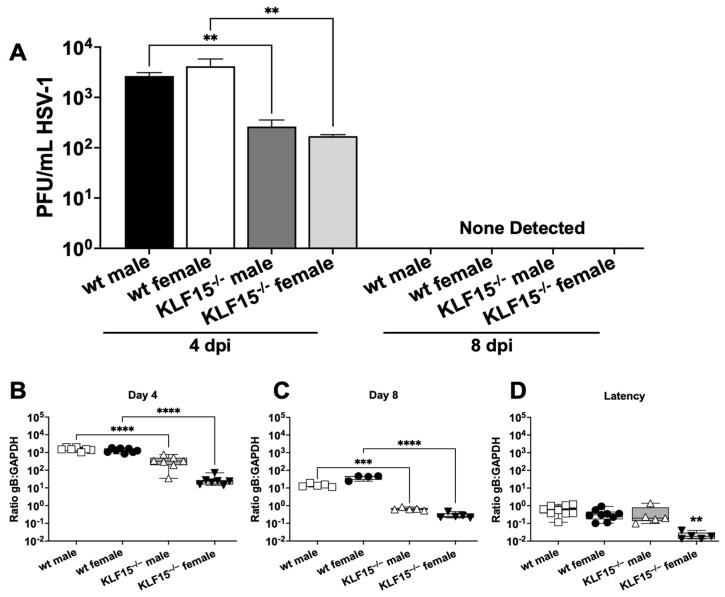
Measurement of infectious virus in mouse TG during acute infection. (**A**): At 4- and 8 days post-infection, TG were dissected from mice (n = 5), minced, and incubated with Vero cell monolayers followed by plaque assays. Data are shown as mean ± SEM for triplicate wells of duplicate experiments. (**B**–**D**): TG from 4–5 animals/group were collected at 4-, 8-, or 30-days post-infection (latency, 2 separate experiments). DNA was prepared from the respective samples, and qPCR was performed using primers for HSV-1 gB or mouse GAPDH. The ratio of gB–GAPDH DNA was calculated using the delta–delta CT method. Data are shown as box-and-whisker plots from max to min, with a line at the mean. ** *p* < 0.01; *** *p* < 0.005; **** *p* < 0.0001 using one-way ANOVA with unpaired multiple comparison post-test.

TG neurons are a crucial site for establishing HSV-1 latency following infection of the ocular cavity, as reviewed in [23,24,25,26]. TG from wt and KLF15^−/−^ male and female mice were collected at 4 and 8 dpi and used to test for virus replication. At 4 dpi, wt male and female TG were shedding ~5000 PFU/mL (Figure 4A). In contrast, the infectious virus present in TG from male and female KLF15^−/−^ mice was significantly lower and contained less than 250 PFU/mL. By 8 dpi, virus shedding from TG of wt and KLF15^−/−^ mice was not detected regardless of sex.

HSV-1 DNA levels in TG were measured by qPCR using primers specific for the highly conserved HSV-1 glycoprotein B [51,52]. TG collected from male and female KLF15^−/−^ mice 4 dpi had significantly lower ratios of HSV-1 gB DNA relative to mouse GAPDH DNA compared to wt mice, with ratios of 500 for KLF15^−/−^ males and only 25 for KLF15^−/−^ females (Figure 4B). In comparison, wt male and female TG had gB–GAPDH ratios greater than 1500. The trend of KLF15^−/−^ mice containing lower HSV-1 DNA in TG was also present at 8 dpi when wt gB–GAPDH ratios dropped to ~25 for male and females. However, KLF15^−/−^ male and female gB–GAPDH ratios were significantly decreased when compared to wt mice (Figure 4C).

By 30 dpi (operationally defined as latency), KLF15^−/−^ DNA ratios (gB versus GAPDH) were significantly lower in female mice versus males (Figure 4D). DNA levels in wt male and female mice were significantly higher than female KLF15^−/−^ mice but not male KLF15^−/−^ mice (Figure 4D). These studies revealed that the establishment of latency, as judged by HSV-1 DNA in TG 30 dpi, was similar for wt males, wt female mice, and KLF15^−/−^ male mice. Conversely, female KLF15^−/−^ mice contained significantly lower levels of viral DNA compared to the other groups.

### 3.5. Analysis of LAT RNA in Latently Infected wt and KLF15^−/−^ Female Mice

**Figure 5:**
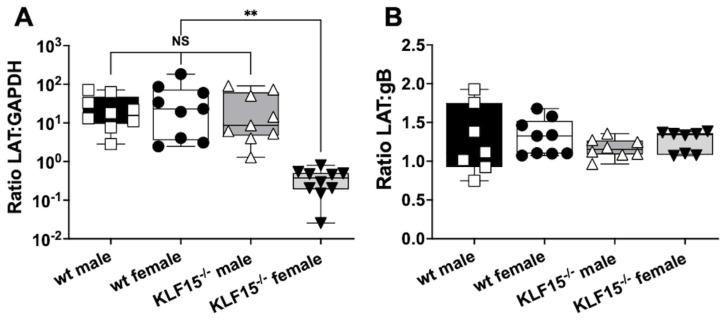
LAT expression in TG of latently infected mice. (**A**): At 30 dpi, TG from latently infected mice were dissected from 5–6 mice/group from 2 separate experiments and then incubated in TRIzol. RNA was prepared as described in the Materials and Methods section. cDNA was synthesized using random hexamers, and qPCR was performed using primers for HSV-1 LAT and mouse GAPDH. (**B**): Cq values for LAT expression (RT-qPCR) were compared to Cq values from gB (qPCR) to calculate the ratio of LAT expression to viral genomes. Data are shown as box-and-whisker plots from max to min with a line at the median for 2 separate experiments (n = 3–5 mice each). ** *p* < 0.001 using one-way ANOVA with unpaired multiple comparison post-test. NS: not significant.

Given the differences in gB–GAPDH DNA ratios for female KLF15^−/−^ mice at 30 dpi compared to male KLF15^−/−^ and wt mice of both sexes, LAT expression was measured. LAT is the only abundantly expressed viral gene during HSV-1 latency and accordingly serves as a hallmark of latency [21,23,24,53]. TG from the respective groups was collected at 30 dpi and RNA prepared for RT-qPCR with primers directed toward LAT (Figure 5A). As expected, LAT levels relative to GAPDH were equal for wt mice (male and female) and KLF15^−/−^ male mice. In contrast, LAT RNA levels were significantly reduced (2-log) for female KLF15^−/−^ mice relative to wt mice and KLF15^−/−^ male mice. When comparing levels of HSV-1 gB DNA to LAT RNA expression, all groups had ratios of approximately 1 (Figure 5B), indicating that LAT expression levels were the same when compared to viral DNA in TG of latently infected mice (Figure 4C). This study also revealed that female KLF15^−/−^ mice contained similar levels of LAT/viral genomes. In summary, these studies suggest that fewer TG neurons in female KLF15^−/−^ mice were latently infected or fewer viral genomes were present in latent neurons.

### 3.6. Analysis of Explant-Induced Reactivation from Latency

**Figure 6:**
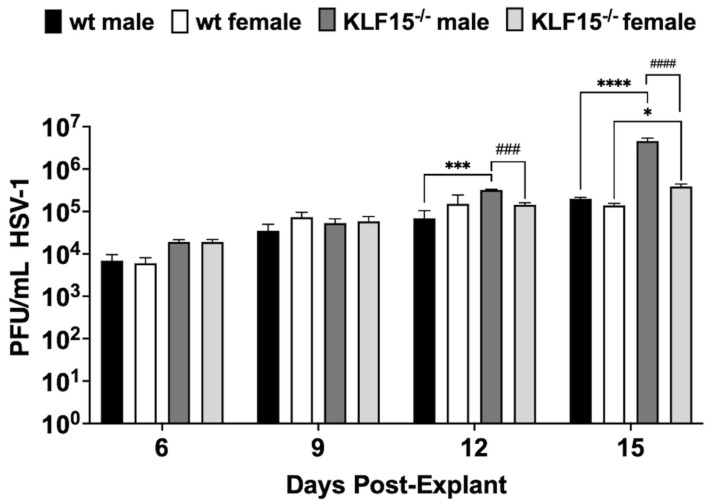
Examination of HSV-1 explant-induced reactivation. TG from male or female wt and KLF15^−/−^ mice (n = 5 mice/group and 3 independent experiments) were dissected at 30 dpi (latency). TG was subsequently minced and incubated in MEM + 2% stripped FBS and 10 µM DEX. Aliquots of supernatant were collected as denoted and used to measure virus titers during HSV-1 explant-induced reactivation from latency. Data are shown as mean ± SEM; * *p* < 0.05; *** *p* < 0.005; **** *p* < 0.0001 for wt vs. KLF15^−/−^; ^###^ *p* < 0.005; ^####^ *p* < 0.001 male vs. female using one-way ANOVA with unpaired multiple comparison post-test.

To investigate HSV-1 reactivation in latently infected KLF15^−/−^ mice, TG were dissected at 30 dpi and explanted in minimal essential medium (MEM) + 2% charcoal-stripped fetal bovine serum (FBS) in the presence of 10 µM DEX to accelerate virus reactivation [28]. Passing FBS through an activated charcoal column removes hormonal factors and reduces the concentration of certain growth factors, vitamins, and metabolites while leaving salts, glucose, and most amino acids important for cellular growth [54,55,56]. Following TG explant, aliquots were collected after explant and used to plaque for the infectious virus. Under the conditions of these studies, virus shedding is not readily detected until 4–6 days post-explant (dpe) [47,48]. All groups exhibited titer increases until 9 dpe with no significant differences (Figure 6). Between 12–15 dpe, virus shedding in TG of wt male and female mice was not significantly increased, indicating that virus production plateaued. Surprisingly, KLF15^−/−^ male mice exhibited a significant increase in virus shedding relative to wt males at 12 or 15 dpi. By 15 days after explant, KLF15^−/−^ male mice had over a 1-log increase in virus replication compared to wt mice regardless of sex. Conversely, TG from female KLF15^−/−^ mice was not significantly different than wt females and males. As in wt mice 12 and 15 days after TG explant, KLF15^−/−^ female TG shed significantly less virus than KLF15^−/−^ males. For example, KLF15^−/−^ male mice shed more than 3-fold HSV-1 compared to KLF15^−/−^ female mice (males shed: ~3 × 10^5^ PFU/mL vs. females shed: ~10^5^ PFU/mL) at 12 dpi. By 15 days after explant, KLF15^−/−^ males shed more than 10^6^ PFU/mL, whereas KLF15^−/−^ female mice remained significantly lower at ~4 × 10^5^ PFU/mL. Despite lower levels of viral DNA in TG of KLF15^−/−^ female mice, there was not a significant reduction in virus shedding compared to wt mice during explant-induced reactivation.

## 4. Discussion

These studies revealed that HSV-1 replication was impaired in ocular swabs (Figure 3B) during acute infection. Furthermore, lower levels of HSV-1 virus shedding in TG were detected at 4- and 8-days post-infection regardless of sex (Figure 4A) whereas lower levels of viral DNA were detected only in TG of female KLF15^−/−^ mice (Figure 4D). A previous study revealed silencing KLF15 in Neuro-2A cells reduced viral replication [43]. Since several studies demonstrated that GR and KLF15 cooperatively transactivate HSV-1 immediate early promoter activity (ICP0, ICP4, and ICP27) [39,40,41,42], these results were expected. Conversely, the finding that HSV-1 grew as efficiently in primary kidney cells from KLF15^−/−^ mice and wt C57Bl/6 mice (Figure 2) was unexpected. We predict that primary kidney cells express certain cellular transcription factors and/or signaling pathways that compensate for lack of KLF15 expression. There are eighteen human KLF family members, suggesting one or more of these proteins compensated for lack of KLF15 expression [31,32,33].

Notably, sex-dependent differences were observed in KLF15^−/−^ mice during the establishment of latency and DEX-induced explant-induced reactivation from latency in TG. For example, male KLF15^−/−^ mice contained higher levels of virus shedding for longer times after TG explant (12 and 15 days after explant) when compared to KLF15^−/−^ females and wt mice regardless of sex. Conversely, female KLF15^−/−^ mice contained lower levels of LAT and HSV-1 DNA levels at 30 dpi when compared to KLF15^−/−^ male mice or wt mice. Despite LAT levels being lower in female KLF15^−/−^ mice, the ratio of LAT compared to viral DNA was not significantly different than wt mice or male KLF15^−/−^ mice. This may be important because LAT plays an essential role in maintaining latency by suppressing lytic gene expression and apoptosis [22,23,24,57]. Based on the observations in KLF15^−/−^ mice, we suggest LAT promotes the establishment and maintenance of latency in a small pool of latently infected TG neurons.

A previous study compared viral replication and reactivation from latency using a mouse model that contains a serine 229 to alanine mutation in GR (GR^S229A^) [48]. Serine 229 in the mouse GR must be phosphorylated for optimal GR-mediated transcription [58]. As in KLF15^−/−^ mice, HSV-1 shedding from cornea and conjunctiva of infected GR^S229A^ mice stopped prior to wt mice. In contrast to KLF15^−/−^ mice, viral replication was significantly reduced in kidney cells from GR^S229A^ mice when compared to wt mice. Furthermore, viral DNA levels in TG were not significantly different in GR^S229A^ and wt mice during latency. As in KLF15^−/−^ mice, HSV-1 viral titers during explant-induced reactivation were significantly reduced in female GR^S229A^ mice versus male GR^S229A^ mice or wt mice. To test whether a mouse that expresses the GR^S229A^ protein but not KLF15, these two mutant mouse strains were bred to produce a double knockout. Double knockout pup frequencies were significantly less than the expected 6.25% [59]. Very few mice survived beyond 10 days postnatal; hence, this approach was abandoned. Since the GR and KLF15 feed-forward transcription loop is important for regulating brain and heart development [35,60,61], and amino acid, lipid, and glucose metabolism [34,44,62], we suggest certain functions of GR and KLF15 expression are necessary for growth and mouse development after birth. Furthermore, GR and KLF15 cooperate to regulate growth and development of skeletal muscle, and transcriptional regulation following corticosteroid-induced muscle atrophy was more profound in male mice [63]. Collectively, these KLF15 functions may explain the observed breeding and developmental differences observed between male and female KLF15^−/−^ mice.

Despite reduced viral replication in male KLF15^−/−^ mice during acute infection, it was surprising to find that virus shedding increased during the late stages of reactivation from latency in male KLF15^−/−^ mice. We suggest other KLF family members or male-specific signaling pathways compensate for the lack of KLF15 during late stages of explant-induced reactivation from latency. Recent studies revealed KLF15 transcriptionally activates expression of Serpina6, which encodes the corticosteroid-binding globulin [64]. In KLF15^−/−^ mice, corticosteroid-binding globulin levels are 90% lower than in wt mice. Consequently, corticosterone levels are 3–4-fold higher in plasma of KLF15^−/−^ mice, which may have allowed viral replication during late stages of reactivation from latency in male KLF15^−/−^ mice. Furthermore, Serpina6 regulates a systemic response to inflammatory stress and KLF15 plays a role in clearing reactive oxygen species. We suggest multiple functions of KLF15 in mammals contributed to the unexpected effects on the HSV-1 latency-reactivation cycle in KLF15^−/−^ mice. Future studies will test whether de novo HSV-1 protein expression during stress-induced reactivation and KLF15 interact with other hormone receptors to provide insight into how KLF15 regulates viral replication and reactivation from latency. In addition to GR and KLF15 cooperatively transactivating the ICP0 promoter [40], ICP4 CRM sequences [41], and ICP27 CRM sequences [42], it is reasonable to predict that other important viral promoters are transactivated by GR and KLF15.

## 5. Conclusions

In conclusion, this research revealed that KLF15 expression is important during acute infection of mice but less important during explant-induced reactivation from latency. For example, despite reduced establishment of latency, explant-induced reactivation from latency was not dramatically affected in female KLF15^−/−^ mice. The finding that male KLF15^−−/−^ mice, but not wt mice or female KLF15^−/−^ mice, continued to shed high levels of virus at 12 and 15 days after explant implies that male-specific transcription factors or signaling pathways maintained reactivation or enhanced the virus spread to adjacent TG neurons and viral replication occurred. Since KLF15 belongs to a large family of transcription factors [64,65,66,67], certain KLF family members may compensate for the lack of KLF15 expression. In addition to DEX activating GR, KLF15 can cooperate with the progesterone receptor and/or the androgen receptor to activate bovine herpesvirus (BoHV-1) immediate early gene expression, which enhances productive infection [68,69,70]. Hence, the progesterone receptor or androgen receptor may play a role in the sex-dependent HSV-1 effects that were observed during acute ocular infection and/or explant-induced reactivation in KLF15^−/−^ mice. Since VP16 specifically transactivates IE promoters and is proposed to trigger reactivation from latency, as reviewed in [23,24,25], it is reasonable to suggest that GR and KLF family members transactivate VP16 promoter activity. However, a VP16 CRM reporter construct was not transactivated by GR and KLF4 or KLF15 [70]. Finally, it is reasonable to predict that male-specific factors prolong the late effects that drove increased virus shedding in male KLF15^−/−^ mice.

## Data Availability

In accordance with MDPI’s data policies, the data presented in this study are available upon request from the corresponding author. Raw data supporting the findings of this research will be made available in compliance with the journal’s guidelines and ethical standards for data sharing.

## Abbreviations

The following abbreviations are used in this manuscript: TG (trigeminal ganglia); DEX (dexamethasone); HSV-1 (Herpes simplex virus-1); GR (glucocorticoid receptor); KLF15 (Krüppel-like factor 15); ICP (infected cell protein); wt (C57BL/6J mice); LAT (latency-associated transcript); PCR (polymerase chain reaction); RT-qPCR (reverse transcription quantitative PCR); PFU (plaque-forming units); dpi (days post-infection); dpe (days post-explant); MOI (multiplicity of infection); min (minutes); bp (base pair); SD (standard deviation); SEM (standard error of the mean); mL (milliliters); gB (glycoprotein B); GAPDH (Glyceraldehyde-3-phosphate dehydrogenase); ANOVA (analysis of variance); BoHV-1 (bovine herpesvirus-1).
